# The two-stage process in visual working memory consolidation

**DOI:** 10.1038/s41598-020-70418-y

**Published:** 2020-08-11

**Authors:** Chaoxiong Ye, Tengfei Liang, Yin Zhang, Qianru Xu, Yongjie Zhu, Qiang Liu

**Affiliations:** 1 Institute of Brain and Psychological Sciences, Sichuan Normal University, Chengdu, China; 2 Department of Psychology, University of Jyvaskyla, Jyvaskyla, Finland; 3 Research Center of Brain and Cognitive Neuroscience, Liaoning Normal University, Dalian, China; 4 Faculty of Information Technology, University of Jyvaskyla, Jyvaskyla, Finland

**Keywords:** Working memory, Human behaviour

## Abstract

Two hypotheses have been proposed to explain the formation manner for visual working memory (VWM) representations during the consolidation process: an all-or-none process hypothesis and a coarse-to-fine process hypothesis. However, neither the all-or-none process hypothesis nor the coarse-to-fine process hypothesis can stipulate clearly how VWM representations are formed during the consolidation process. In the current study, we propose a two-stage process hypothesis to reconcile these hypotheses. The two-stage process hypothesis suggests that the consolidation of coarse information is an all-or-none process in the early consolidation stage, while the consolidation of detailed information is a coarse-to-fine process in the late consolidation stage. By systematically manipulating the encoding time of memory stimuli, we asked participants to memorize one (Experiment 1) or two (Experiment 2) orientations in different encoding time intervals. We found that the memory rate increased linearly as the encoding time increased. More importantly, VWM precision remained constant when the encoding time was short, while the precision increased linearly as the encoding time increased when the encoding time was sufficient. These results supported the two-stage process hypothesis, which reconciles previous conflicting findings in the literature.

## Introduction

We need to rely heavily on visual information to meet the needs of serial cognitive tasks^1^. The visual stimulus of the external world can be transferred to perception representations. However, perception representation is unstable and susceptible to interference from new information, so it needs to be transformed into another stable form of visual information. This new form of information is visual working memory (VWM, also known as short-term memory) representation, and the process of forming memory representation is called VWM consolidation^2^.

Recent studies on the consolidation of VWM have investigated the time course of consolidation^2,3^, the bandwidth of consolidation^4–6^, and the difference in the consolidation mechanisms of various visual features^7–9^. For example, by presenting post masks immediately after the disappearance of memory stimuli, researchers manipulated the encoding time of participants for memory stimuli, thereby indirectly controlling the time allowed for VWM consolidation^4–10^. However, a consensus has not yet been reached on the formation manner for VWM representations during the consolidation process. Two hypotheses have been proposed: an all-or-none process hypothesis and a coarse-to-fine process hypothesis. The all-or-none hypothesis suggests that, when the perception representation is consolidated to VWM representation, the full representation will be created directly but, if the encoding time is not sufficient, the consolidation process will fail^11^. Conversely, the coarse-to-fine hypothesis suggests that the formation of creating VWM representations is a process of transition from rough representations to high-precision representations^12^.

Previous studies have supported both the all-or-none and coarse-to-fine hypotheses. For example, Zhang and Luck^11^ manipulated the consolidation time of memory stimuli and found that decreasing the consolidation time interval produced a large decline in memory rate but no change in memory precision, thus supporting the all-or-none hypothesis. However, Gao et al.^12^ manipulated the encoding time interval and found that low-precision information preceded high-precision information when entered into VWM, thus supporting the coarse-to-fine hypothesis. Nevertheless, the all-or-none and coarse-to-fine hypotheses need not be mutually exclusive. The contradiction between the two hypotheses may be caused by the different encoding/consolidation time intervals chosen by previous studies. For example, the study supporting the all-or-none hypothesis used a relatively short encoding/consolidation time interval (113 ms/item with post masks)^11^, while the study supporting the coarse-to-fine hypothesis chose a relatively long encoding/consolidation time interval (125–250 ms/item without post masks)^12^. Therefore, the interval length of the encoding/consolidation time may affect the formation manner of representation.

The formation manner of representation could be explained based on the framework of a recent model for explaining resource allocation in VWM consolidation, called the two-phase resource allocation model^13,14^. The two-phase resource allocation model suggests that whether there is sufficient encoding time before the stimulus is removed or displaced affects the resource allocation in VWM. Two different phases are included in the consolidation of VWM representation: the early consolidation phase and the late consolidation phase. In the early consolidation phase, individuals create representations of minimum precision for all items of the memory array. Individuals consolidate as many items as possible to ensure that they can remember more visual information. The duration of the early phase increases with the number of memory items. Thus, in this phase, individuals can only involuntarily allocate VWM resources to all items in a stimulus-driven manner. After completing the early phase, if the visual information still exists, individuals can continue the late consolidation phase. In the late phase, individuals can voluntarily allocate resources according to task demand. Individuals may further allocate the remaining resources or reallocate the resources to improve the precision of the memory representations. This model is also supported by recent studies^15,16^, which suggest that, while the initial resource allocation was independent of the participants’ intentions and the importance of memory items, more controlled mechanisms reallocate resources when the encoding time is sufficient.

According to the framework of the two-phase resource allocation model, we proposed a two-stage process hypothesis to explain the formation manner for VWM representations. The two-stage process hypothesis suggests that the all-or-none and coarse-to-fine hypotheses are not mutually exclusive and that the formation of VWM representation includes two different stages. The consolidation of coarse information is an all-or-none process in the early consolidation stage. In this stage, the precision of VWM representation remains constant at a low-precision level, but does not change as the encoding time increases. After individuals complete the early stage,  in the late consolidation stage, the consolidation becomes a coarse-to-fine process. In the late stage, the precision of VWM representations increases with the encoding time. Moreover, when the consolidation is a serial process (e.g., for orientation, see^5,6^), the duration of the early consolidation stage (all-or-none process) is extended as memory set size increases. It is worth noting that the purposes of the two-phase resource allocation model and the two-stage process hypothesis are different. The two-phase resource allocation model explains how individuals allocate VWM resources, while the two-stage process hypothesis explains how VWM representations form.

Although there have been some previous studies on the formation of VWM representation^11,12^, more studies need to investigate this topic due to a potential problem observed in previous studies: the selection of the encoding/consolidation time of memory stimuli. Firstly, the number of time interval conditions of previous studies on the formation manner of VWM representations, which chose only a few time intervals (i.e., two), might not be enough to manipulate the encoding/consolidation time. Secondly, although a previous study has shown that there were large distinct differences in consolidation ability among individuals^17^, previous studies on representation formation often overlooked these individual differences as related to the speed of VWM consolidation. If a two-stage process forms VWM representation, choosing fixed encoding/consolidation time intervals for all participants may lead to different participants entering different consolidation stages. In order to mitigate this problem, recent studies on consolidation used a thresholding procedure to manipulate the encoding time of memory stimuli by calculating a duration that produced the same accuracy for each participant^4,5,7,9^.

Another study suggested that the limitations of processing resources would rise when presenting all memory stimuli simultaneously compared to sequential presentation^18^, however, previous studies on representation formation often asked participants to consolidate multiple items simultaneously^11^. In order to reduce the cost of resource competition caused by simultaneous consolidation, it is necessary to investigate the formation of representation by asking participants only to consolidate a single item.

Experiment 1 tested the two-stage process hypothesis when the memory set size was one. We asked participants to memorize one orientation in an orientation recall task and systematically manipulated the encoding time of memory stimulus based on their consolidation speed. The consolidation speed of each participant was measured by a pretask of thresholding procedure, which has been used widely in recent consolidation studies^4,5,7,9^. By fitting the response error data of each participant with a standard mixture model^11^, we can observe memory precision and memory rate at different encoding time intervals. The encoding time of the memory stimulus was defined as the time interval when the participants could encode/consolidate memory items effectively. The two-stage process hypothesis predicts that, when the encoding time is less than the time interval required for consolidating one item, memory precision will remain unchanged. However, when the encoding time is longer than the time interval required for consolidating one item, memory precision will increase along with the encoding time.

## Experiment 1

### Methods

#### Participants

Nineteen students from Liaoning Normal University (4 male and 15 female, 19–26 years old with a mean age of 22.31 years) volunteered to participate in this experiment for compensation at a rate of $3/h. They reported having normal color vision and normal or corrected-to-normal visual acuity, with no history of neurological problems. Each participant provided written informed consent before the experiment. All procedures were conducted following the Declaration of Helsinki (2008) and were approved by the ethics committee of Liaoning Normal University.

#### Visual Stimuli

The memory stimuli were the same as those used in previous studies on VWM consolidation of our group^5,6,9,13:^ sinusoidal gratings (contrast, 0.7; spatial frequency, three cycles/deg) in a circular aperture (size, 0.9°) presented on a gray background. The masks were circular apertures (size, 1°) containing pixel noise with random luminance. The orientation of each stimulus was randomly selected from 90 possible angles spaced evenly from 0° to 180°, with the orientations separated by at least 12°. The gratings were presented at one of four possible locations located at the corners of an imaginary square (eccentricity, 3°). The stimuli were presented on a 19-inch CRT monitor (1,280 × 768 pixel). The distance between the monitor and the participant was about 60 cm.

### Procedures

#### Pretest task: thresholding procedure

**Figure 1:**
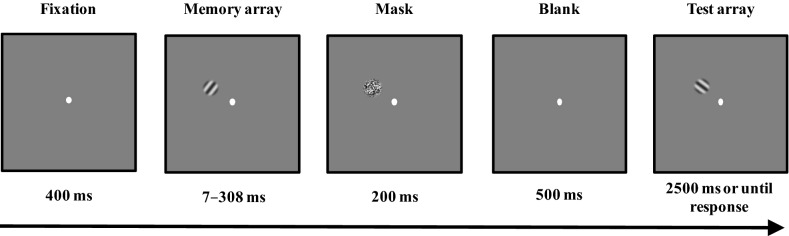
Trial structure of the thresholding procedure in Experiment 1.

We used the same thresholding procedure as that used in previous studies^7,9^. Before participating in the main task, all participants performed a pretask: an orientation change detection task with the trial structures depicted in Fig. 1. At the beginning of each trial, a fixation was presented in the middle of the screen. Then, an orientation stimulus was presented for eight possible encoding time intervals (7 ms, 14 ms, 28 ms, 56 ms, 98 ms, 154 ms, 224 ms, and 308 ms). Participants were asked to stare at the fixation and memorize the orientation. After the orientation stimulus disappeared, a mask was presented for 200 ms. Followed by a blank interval of 500 ms, a test item of one orientation stimulus was presented. The participants’ task was to indicate whether the test item was identical to the memory array. After their responses, a blank period of 600–700 ms preceded the onset of the next trial. The test item was different from the memory item in 50% of trials, while it was identical to the memory item in the rest of the trials. If the test item was different from the memory item, a new orientation with a 90° direction-difference from the memory item was selected for the test item.

The pretask consisted of 40 trials of each time interval condition, with a total of 320 trials. Each participant’s percentage correct was calculated for each interval. The data were fitted with the exponential function

$$pc = \delta + \gamma (1 - e^{ - \beta t} ),$$

where $pc$ stands for the accuracy (percentage correct), $t$ stands for encoding time, and $\delta$, $\gamma$, and $\beta$ are free parameters that control the shape of the psychometric function. The standard maximum likelihood methods were used to fit the data. The time intervals that yielded overall accuracies of ~ 70%, ~ 80%, or ~ 90% correct were used for the encoding time of memory array for different conditions in the main task.

#### Main task

**Figure 2:**
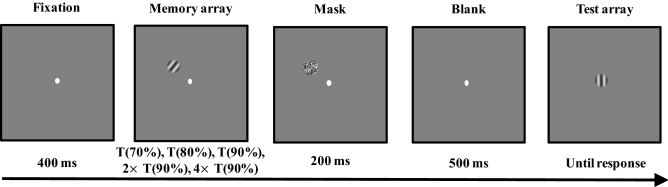
Trial structure of the main task in Experiment 1.

Participants completed an orientation recall task during the main task. The basic trial structure is shown in Fig. 2. At the beginning of each trial, a fixation was presented at the center of the screen. Then, a memory item of orientation was presented in five encoding time conditions (corresponding time of ~ 70% correct [T(70%)], corresponding time of ~ 80% correct [T(80%)], corresponding time of ~ 90% correct [T(90%)], twice the corresponding time of ~ 90% correct [2 × T(90%)] and quadruple the corresponding time of ~ 90% correct [4 × T(90%)]) and followed by a mask (200 ms). The corresponding time of different accuracies was calculated by the threshold procedure described above. After a blank screen was presented for 500 ms, a test item of an adjustable orientation was presented at fixation. Participants were asked to stare at the fixation, remember the orientation of the memory item, and use the mouse to adjust the orientation of the test item to match that of the memory item. After their responses, a blank period of 600–700 ms preceded the onset of the next trial.

There were 100 trials for each encoding time condition, which were fully randomized, with a total of 500 trials. At least 20 practice trials were run to help the participants understand the instructions. The entire experiment lasted approximately 60 min.

#### Data Analysis

For each trial, we calculated the errors in the reported orientation by subtracting the orientation of the adjusted test item from that of the memory item. By using the MemToolbox^19^, we fit the error data with the standard mixture model^11^. The standard mixture model assumed that participants’ responses could be divided into two types of trials. In some of the trials, participants did not consolidate the orientation into VWM but guessed, with the reported orientation conforming to a uniform random distribution. In the remaining trials, participants successfully consolidated the orientation into VWM, which contained a noisy representation of the target orientation, modeled by a von Mises distribution. The standard mixture model allowed us to estimate the guess rate (*P*_*g*_) as well as the precision of VWM representation (*SD*). The *P*_*g*_ was the proportion of guess trials in all trials and was inversely related to memory rate. *SD* was the circular standard deviation of a von Mises distribution, which was inversely related to VWM precision. We fitted the standard mixture model to individual participant data in each condition.

A repeated measure ANOVA with encoding time as a within-subject factor was conducted for *SD* and *P*_*g*_. With a bootstrapping method (SPSS version 24.0; 10,000 permutations with 95% confidence intervals), the follow-up paired t-tests were conducted for the comparison of different duration conditions for *SD* and *P*_*g*_. Cohen’s *d* was reported as the effect size for the t-tests. The results of the Bayes factor analysis were also reported^20^. Bayes factors (*BF*_*10*_) can provide an odds ratio for the alternative/null hypotheses (*BF*_*10*_ > 1 favor the alternative hypothesis and *BF*_*10*_ < 1 favor the null hypothesis). For example, a *BF*_*10*_ of 0.5 indicates that the null hypothesis is two times more likely than the alternative hypothesis.

### Results

**Figure 3:**
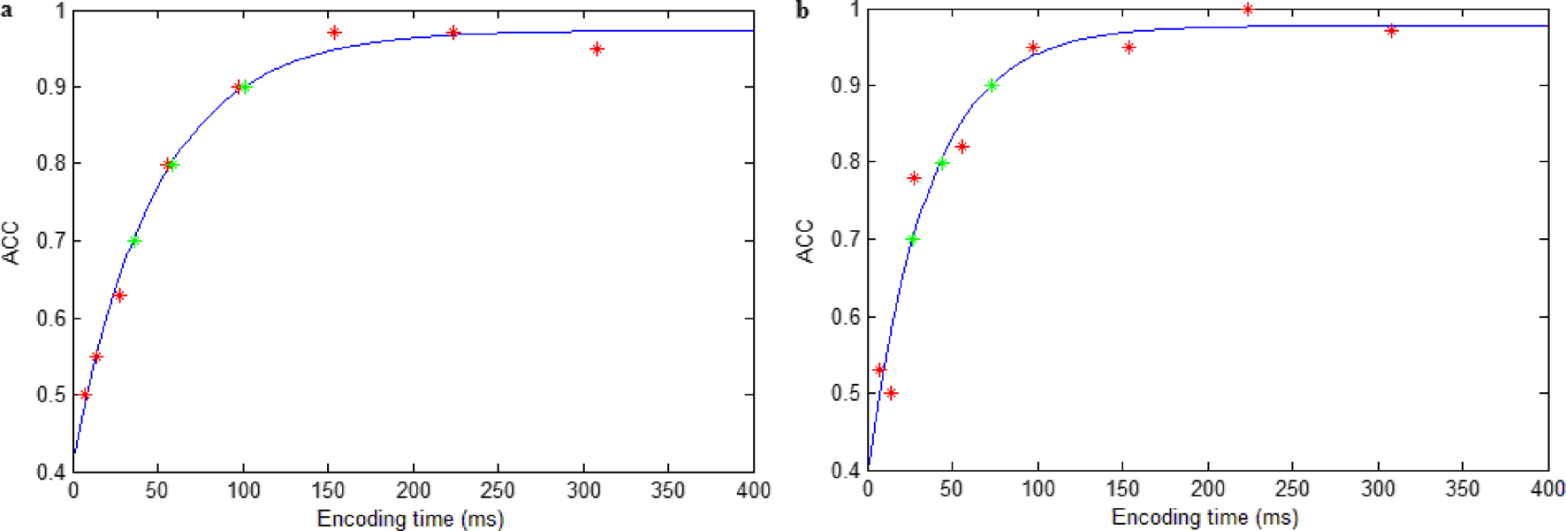
Results of fitting the psychometric function to samples in Experiment 1 (**a**) and 2 (**b**). The red stars show the accuracy at different encoding time intervals in the pretest task. The continuous blue lines illustrate the function fits. The green stars show the expected duration that produced ~ 70%, ~ 80%, and ~ 90% correct.

The average T(70%) across participants was 29.47 ms (range = 7–98 ms, *SD* = 20.55 ms), the average T(80%) was 50.11 ms (range = 14–147 ms, *SD* = 31.24 ms), and the average T(90%) was 91.37 ms (range = 28–238 ms, *SD* = 54.15 ms). Figure 3a shows the results of fitting the psychometric function to a sample.

**Figure 4:**
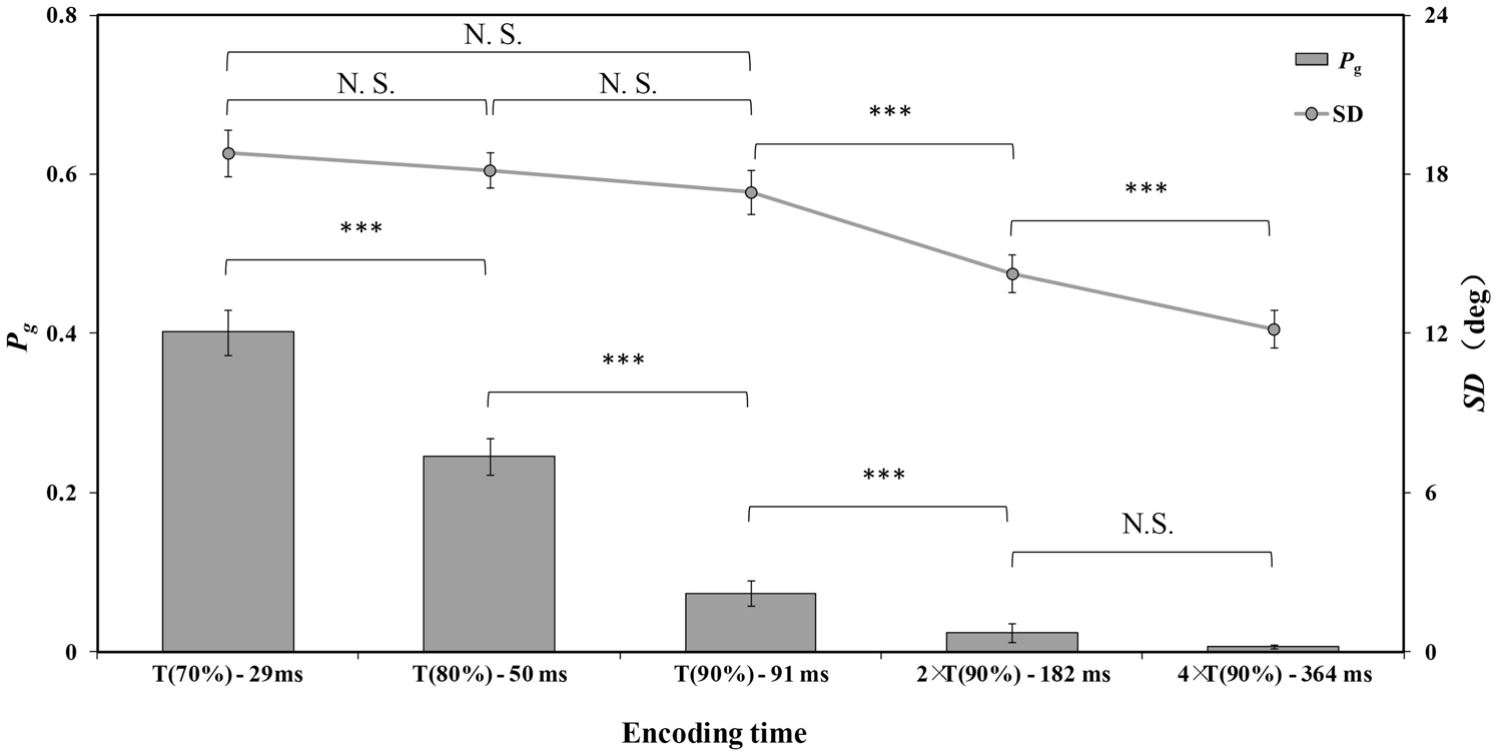
The mean precision parameter (SD) and guess-rate parameter (Pg) for each encoding time condition in Experiment 1. The abscissa axis represents the average encoding time of all participants in each condition. The error bars represent standard error. ***p < .001; *N.S.* non-significant.

The results of the precision parameter (*SD*) and guess-rate parameter (*P*_*g*_) for each encoding time condition are shown in Fig. 4. For the precision parameter (*SD*), a one-way repeated measures ANOVA confirmed that encoding time yielded main effects, *F*(4,72) = 26.358, *p* < 0.001, η^2^ = 0.594. Follow-up paired t-tests showed that *SD* did not differ significantly across T(70%), T(80%), and T(90%) conditions (*t*(18) = 0.744, *p* = 0.467, CI_95%_ = [− 1.18, 2.48], Cohen’s *d* = 0.189, *BF*_10_ = 0.304, for T(70%) and T(80%) conditions; *t*(18) = 0.949, *p* = 0.355, CI_95%_ = [− 1.00, 2.65], Cohen’s *d* = 0.249, *BF*_10_ = 0.353, for T(80%) and T(90%) conditions; *t*(18) = 1.629, *p* = 0.121, CI_95%_ = [− 0.43, 3.37], Cohen’s *d* = 0.394, *BF*_10_ = 0.726, for T(70%) and T(90%) conditions), but the *SD* in the T(90%) condition is significantly higher than that in the 2 × T(90%) condition, *t*(18) = 6.654, *p* < 0.001, CI_95%_ = [2.10, 4.04], Cohen’s *d* = 0.919, *BF*_10_ > 100. In addition, *SD* in the 2 × T(90%) condition is significantly higher than that in the 4 × T(90%) condition, *t*(18) = 5.274, *p* < 0.001, CI_95%_ = [1.26, 2.93], Cohen’s *d* = 0.686, *BF*_10_ > 100.

For the guess-rate parameter (*P*_*g*_), a one-way repeated measures ANOVA confirmed that encoding time yielded main effects, *F*(4,72) = 105.547, *p* < 0.001, η^2^ = 0.854. Follow-up paired t-tests showed that *P*_*g*_ linearly reduced with the increase of encoding time from T(70%) to 2 × T(90%) (*t*(18) = 5.139, *p* < 0.001, CI_95%_ = [0.09, 0.22], Cohen’s *d* = 1.381, *BF*_10_ > 100 for T(70%) and T(80%); *t*(18) = 8.488, *p* < 0.001, CI_95%_ = [0.13, 0.22], Cohen’s *d* = 2.024, *BF*_10_ > 100, for T(80%) and T(90%); *t*(18) = 3.745, *p* < 0.001, CI_95%_ = [0.02, 0.07], Cohen’s *d* = 0.832, *BF*_10_ = 26, for T(90%) and 2 × T(90%), but that *P*_*g*_ in the 2 × T(90%) condition did not differ significantly from that in the 4 × T(90%) condition, *t*(18) = 1.705, *p* = 0.105, CI_95%_ = [− 0.01, 0.04], Cohen’s *d* = 0.469, *BF*_10_ = 0.803.

### Discussion

The results of Experiment 1 show that the manipulation of encoding time did not affect memory precision (i.e., *SD*^−*1*^) when the encoding time was between T (70%) and T (90%), but that memory rate (i.e., 1 − *P*_*g*_) increased linearly with the encoding time. When the encoding time increased from T(90%) to 4 × T(90%), memory precision increased linearly, but memory rate reached a ceiling value when the encoding time was 2 × T(90%). The null result for memory rate between the 2 × T(90%) condition and 4 × T(90%) condition could be due to the floor effect of guess rate (lower than 2.5%) at long encoding time intervals. However, memory precision did not increase when the encoding time increased from T(70%) to T(90%). These results did not support the coarse-to-fine hypothesis. Our results also rejected the all-or-none hypothesis because the precision increased as the encoding time increased from T(90%) to 4 × T(90%). However, the results supported the two-stage process hypothesis, which suggests that the representation was formed as a hybrid of both all-or-none and coarse-to-fine manners.

The two-stage process hypothesis could be explained by the frameworks of both discrete resource theory^11,21–24^ and continuous resource theory^25–28^, which are two broad theories proposed for the nature of VWM. In the framework of the discrete resource theory, the recent slots + averaging model suggests that VWM has a limited number of available discrete resources, like “slot”^11^. In general, humans have about 3–4 slots. Only the representations allocated slots can be maintained in VWM. Multiple slots can be allocated to a representation to improve memory precision further. Becker et al.^5^ used the same stimuli as the present study and found that the consolidation of orientations was a serial process, which means that only one orientation can be consolidated into VWM at a time. Based on the slots + averaging model, VWM consolidation is a process of allocating slots to memory items one by one. When the encoding time was between T(70%) and T(90%), participants allocated the first slot to the memory representation. An all-or-none manner created coarse representation in the early consolidation stage. The probability of successfully allocating slots increased as the encoding time increased in this period, but only one slot could be assigned to the memory representation. Thus memory precision of VWM representation was at the minimum level (i.e., one slot). There was no change in memory precision while memory rate was linearly increasing; however, when the encoding time was between T(90%) and 4 × T (90%), participants had finished the all-or-none process and started the coarse-to-fine process in the late consolidation stage: they could allocate more slots to the same memory representation. Thus, memory precision increased with the encoding time. When multiple slots represented an item, the possibility of losing the item’s memory representation was further reduced, which could explain why memory rate increased with the encoding time when the encoding time was longer. Although the results seem to favor the discrete resource theory, they might also be explained by the continuous resource model. In the framework of the continuous resource theory, VWM consists of a pool of flexible resources. Allocating resources to representation is a gradual process. In the early consolidation stage, participants needed time to create VWM representations that could be used to complete the task (minimum precision). After a VWM representation with minimum precision was created, participants could then enter the late stage. In this stage, memory precision was further improved, which could explain why we found that memory rate increased linearly with the encoding time, while memory precision remained constant when the encoding time was short. Therefore, within the frameworks of both discrete resource theory and continuous resource theory, the two-stage process hypothesis could explain the results of Experiment 1. The basic mechanism underlying the two-stage process was that participants could only allocate VWM resources gradually (e.g., slot by slot) as the encoding time increased.

## Experiment 2

In Experiment 1, we tested the two-stage process hypothesis by asking participants to memorize one item, but the representation formation manner for memorizing multiple items remained unclear. Since previous studies have demonstrated that the consolidation of orientations is a serial process^6,9^, based on the results of Experiment 1, there were two different possibilities for the representation formation manner when multiple orientations needed to be consolidated. The first possibility was the item-based serial consolidation hypothesis, which hypothesis assumes that participants serially consolidate orientations. There should be a two-stage process for each item. For example, participants would start to consolidate the second orientation only after they finished the late consolidation stage of the first orientation. The second possibility is the stage-based serial consolidation hypothesis, which assumes that, at the early consolidation stage, by allocating minimum needed resources (e.g., one slot) to each representation, participants would serially consolidate all orientations into VWM to create more low-precision representations (an all-or-none process). When the early consolidation for all orientations was completed, participants would further allocate the remaining resources to each representation to improve memory precision (a coarse-to-fine process).

Experiment 2 further investigated the two-stage process hypothesis by testing the item-based serial consolidation and stage-based serial consolidation hypotheses when the memory set size was two. According to the results of Experiment 1, T(90%) can be considered as the time it took to complete the early consolidation stage for one orientation. In Experiment 2, we asked participants to memorize two orientations and systematically manipulated encoding time in four conditions (T(90%), 2 × T(90%), 4 × T(90%), and 6 × T(90%)). The item-based hypothesis expected memory precision to increase as the encoding time increased from T(90%) to 2 × T(90%), and then that memory precision would remain constant or even decrease as the encoding time increased from 2 × T(90%) to 4 × T(90%). The stage-based hypothesis expected no difference in memory precision between the T(90%) condition and 2 × T(90%) condition but expected memory precision to increase as the encoding time increased from 2 × T(90%) to 4 × T(90%). In order to observe the change in memory rate and memory precision when the encoding time was sufficiently long, we chose the encoding time interval of 6 × T (90%) as a longer encoding time condition.

### Methods

#### Participants

A new sample of seventeen students (8 male and 9 females, 18–25 years old with a mean age of 22.41 years) from Liaoning Normal University volunteered to participate in this experiment for compensation at a rate of $3/hour. They reported having normal color vision and normal or corrected-to-normal visual acuity, with no history of neurological problems. Each participant provided written informed consent before the experiment. The ethics committee of Liaoning Normal University approved all procedures.

#### Visual Stimuli and Procedures

**Figure 5:**
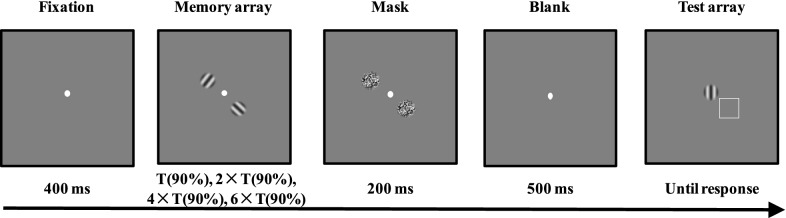
Trial structure of the main task in Experiment 2.

The stimuli and procedures of Experiment 2 were like those in Experiment 1 with the following exceptions: we used the same stimuli as those in Experiment 1, but increased the total number of items in the memory array to two. The memory array was displayed for four encoding time conditions (the corresponding time of ~ 90% correct [T(90%)], twice the corresponding time of ~ 90% correct [2 × T(90%)], quadruple the corresponding time of ~ 90% correct [4 × T(90%)] and sextuple the corresponding time of ~ 90% correct [6 × T(90%)]). The same threshold procedure calculated the corresponding time of ~90% correct as in Experiment 1. In all conditions, when the test array presented, a spatial cue (a 1.2° square outline) appeared along with an adjustable test orientation (presented at the fixation). Participants needed to adjust the orientation of the test item to match that of the cued memory item. There were 160 trials for each encoding time condition, with a total of 640 fully randomized trials. Figure 5 shows the basic trial structure of the main task.

### Results

The average T(90%) across participants was 124.76 ms (range = 49–238 ms, *SD* = 59.25 ms). Figure 3b shows the results of fitting the psychometric function to a sample.

**Figure 6:**
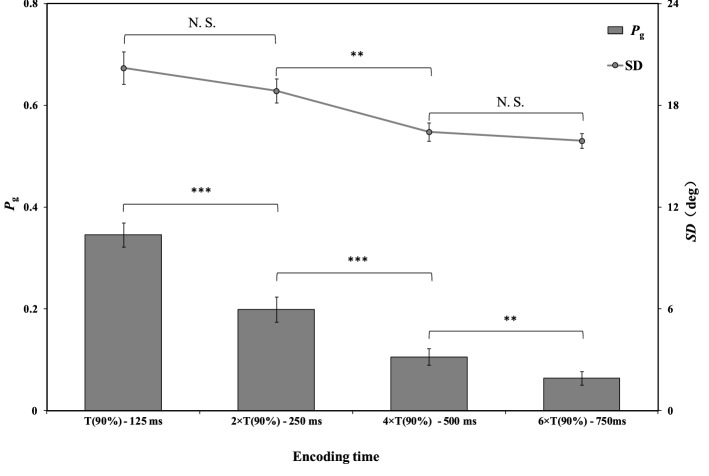
The mean precision parameter (SD) and guess-rate parameter (Pg) for each encoding time condition in Experiment 2. The abscissa axis represents the average encoding time of all participants in each condition. The error bars represent standard error. ***p < .001; **p < .01; *N.S.* non-significant.

The results of the precision parameter (*SD*) and guess-rate parameter (*P*_*g*_) for each encoding time condition are shown in Fig. 6. For the precision parameter (*SD*) , a repeated measures one-way ANOVA confirmed that encoding time yielded significant main effects, *F*(3,48) = 13.219, *p* < 0.001, η^2^ = 0.452. Follow-up paired t-tests showed that the *SD* in the T(90%) condition was no significant difference from that in the 2 × T(90%) condition, *t*(16) = 1.506, *p* = 0.151, CI_95%_ = [− 0.55, 3.25], Cohen’s *d* = 0.388, *BF*_10_ = 0.645. *SD* in the 2 × T(90%) condition was significantly higher than that in the 4 × T(90%) condition, *t*(16) = 3.553, *p* < 0.01, CI_95%_ = [0.97, 3.86], Cohen’s *d* = 0.928, *BF*_10_ = 16.403. *SD* in the 4 × T(90%) condition was no significantly different from that in the 6 × T(90%) condition, *t*(16) = 1.741, *p* = 0.101, CI_95%_ = [− 0.11, 1.17], Cohen’s *d* = 0.259, *BF*_10_ = 0.865.

For the guess-rate parameter (*P*_*g*_), a repeated measures one-way ANOVA confirmed that encoding time yielded main effects, *F*(3,48) = 76.171, *p* < 0.001, η^2^ = 0.826. Follow-up paired t-tests showed that the *P*_*g*_ linearly reduced as the encoding time increased (*t*(16) = 5.340, *p* < 0.001, CI_95%_ = [0.09, 0.21], Cohen’s *d* = 1.484, *BF*_10_ > 100, for T(90%) and 2 × T(90%); *t*(16) = 4.425, *p* < 0.001, CI_95%_ = [0.05, 0.14], Cohen’s *d* = 1.083, *BF*_10_ = 79.60, for 2 × T(90%) and 4 × T(90%); *t*(18) = 3.671, *p* < 0.01, CI_95%_ = [0.02, 0.07], Cohen’s *d* = 0.697, *BF*_10_ = 20.30, for 4 × T(90%) and 6 × T(90%)).

### Discussion

The results of Experiment 2 show that memory precision (i.e., *SD*^−*1*^) did not change between the T(90%) condition and 2 × T(90%) condition, but that precision increased when the encoding time increased from 2 × T(90%) to 4 × T(90%), and remained consistent when the encoding time was between 4 × T(90%) and 6 × T(90%). Memory rate (i.e., 1 − *P*_*g*_) increased linearly as the encoding time increased from T(90%) to 6 × T(90%).

These results support the stage-based serial consolidation hypothesis and reject the item-based serial consolidation hypothesis. For memory precision, when the encoding time was between T(90%) and 2 × T(90%), participants allocated the minimum resources for completing the early stage to each orientation in a serial way. In this stage, only a few resources could be assigned to each representation. Because the memory precision of all VWM representations was at the minimum level (e.g., the one-slot level), memory precision did not increase. When the encoding time was increased from 2 × T(90%) to 4 × T(90%), participants entered the late consolidation stage. More resources could be allocated to each orientation (e.g., two slots), and memory precision was significantly higher than that at the minimum level. However, when the encoding time increased from 4 × T(90%) to 6 × T(90%), the precision of memory representations reached the cell level. Thus, memory precision remained consistent. For memory rate, because more resources were continuously allocated to memory representations, the possibility of forgetting or losing memory representations would further decrease as the encoding time increased. Thus, the memory rate increased as the encoding time increased from T(90%) to 6 × T(90%).

The results of Experiment 2 indicate that participants needed at least 2 × T(90%) to complete the all-or-none process for two orientations. This result was in line with the expectation of the two-stage process hypothesis. These results also suggest that the consolidation time for the all-or-none manner was extended as memory set size increased.

## General discussion

The present study investigated how VWM representations were formed during the consolidation process. We proposed a two-stage process hypothesis to explain the findings of previous studies. In order to test the two-stage process hypothesis, we manipulated the encoding time in a recall task with a mask when participants only needed to consolidate one orientation in Experiment 1. We found that memory precision was stable in the early consolidation stage and increased linearly in the late consolidation stage. However, the memory rate increased linearly with the encoding time until it reached a ceiling level. The two-stage process hypothesis could explain these results based on the frameworks of both the discrete resource and continuous resource theories. In Experiment 2, in order to explore how representations were formed when participants needed to consolidate multiple representations simultaneously, we used procedures similar to those of Experiment 1 but increased the set size of memory orientations from one to two. We found that memory precision was stable when the encoding time was short (early consolidation stage), and increased linearly from twice the encoding time it took to consolidate one orientation. Memory precision reached a ceiling level until quadruple the encoding time it took to consolidate one orientation. However, the memory rate increased linearly with the encoding time. Our findings suggest that the resources were not only serially allocated to all items, but that priority was given based on the quantity of all memory items (stage-based serial consolidation hypothesis) instead of the quality of each item (item-based serial consolidation hypothesis). Our results suggest that individuals allocated the resources gradually to each memory representation in the early consolidation stage. After each representation received minimum resources (e.g., one slot) for completing the early stage, individuals could then allocate the rest of the resources to the consolidated representations to further improve memory precision. This result was also in line with the framework of the two-phase resource allocation model^13^. The two-stage process hypothesis is, therefore, an important complement to existing VWM research.

The results of Experiments 1 and 2 show that the memory rate increased as the encoding time increased. This relationship suggests that longer encoding time intervals could reduce the degree to which the masks interfere with memory representations. With the increase of encoding time, the memory representations became more stable, and it was more difficult for the masks to impair the memory representations that had already been consolidated.

Zhang and Luck^11^ used a more traditional method to manipulate the consolidation time. In their study, the exposure duration of the memory items was fixed (100 ms), and only the SOA (stimulus onset asynchrony of 110 ms or 340 ms) between memory items and post masks was manipulated. The masks appeared 10 ms or 240 ms after the memory items disappeared. In our study, we directly manipulated the exposure duration of the memory items. There was no blank between the offset of the memory items and the onset of the masks. Thus, the exposure duration of the memory item was constant under different conditions in Zhang and Luck^11^’s study, but it was different under different conditions in our study. Two reasons informed our direct manipulation of the exposure duration of memory items. In Zhang and Luck^11^’s study, although the exposure duration of memory items was fixed, visible persistence could still store information for at least 100 to 200 ms after memory items disappeared because of retinal persistence^29–32^. Because there may be large individual differences in the duration of retinal persistence, the participants could continue the encoding process during the blank before the post masks were presented. Although the exposure duration of memory items was fixed, it did not mean that each participant had the same fixed encoding time under the same condition. In our study, however, the exposure duration of memory items was the same as the encoding time. Moreover, our experimental design was based on the serial consolidation of orientation materials. The serial consolidation of orientation information is supported by recent studies that manipulated the exposure duration of memory items^5–7,9^. Our study chose the same orientation stimuli as these previous studies on consolidation, and we used the same method (i.e., no SOA) to manipulate the exposure duration of memory items.

Since we directly manipulated the exposure duration of memory items, the SOAs between memory items and test array were not held constant in our study. The lengths of memory maintenance time may affect VWM performance; for example, a longer memory maintenance process may lead to the decay of VWM representations and thus impair memory performance. However, there are two reasons to reject this possibility. Firstly, because the exposure duration of masks (200 ms) and the blank (500 ms) between masks and test array were fixed, the length of SOAs between memory items and test array was only affected by the exposure duration of memory items (i.e., encoding time). VWM performance under the long encoding time (long SOA) condition was better than that in the short encoding time (short SOA) condition. This result was not consistent with expectations. Secondly, Zhang and Luck^33^’s study had not shown a significant decline in VWM performance when the SOA between memory items and test array was less than 4,000 ms. In our study, the overall range of SOA between the memory items and test array was between 700 ms and 1,500 ms, and the variation in SOAs across different conditions was within 800 ms. Thus, the SOA setting in our study would not lead to significant decay of VWM representations, and the SOAs between memory items and test array under different conditions did not cause potential contamination in our results.

A point worthy of discussion is the relationship between the eye movement of participants and our results. In humans, a saccade takes about 200 ms (or a bit less). Our results show that VWM precision increased most when the encoding time increased from ~ 100 to ~ 200 ms for one orientation(Experiment 1), and from ~ 250 to ~ 500 ms for two orientations(Experiment 2). The eye movement of participants may have contributed to the improvement of VWM precision. The process of visual information during eye movement can be divided into different stages. Before the saccade, individuals first shift attention to the location of the impending saccade targets (the eyes remain at the fixation)^34^, and encode the target items to form coarse VWM representations^26^. After that, saccades towards the targeted items begin. The visual information of the targeted items is maintained in VWM across the saccade, supporting the experience of perceptual continuity. After the saccade, the peripheral visual content is brought to the fovea. Individuals can correct errors of the initial encoding and further encode target items in a highly precise way^35^. Thus, in our study, the precision of VWM representations could substantially increase once participants fulfilled one saccade. The latency of the eye movement effect can be prolonged with the increase of memory set size; that is, the change of the participants’ behavior (especially memory precision) in different encoding time conditions may depend on whether participants had sufficient time to complete the saccade towards the location of the memory targets. Although we asked the participants to stare at the fixation during the task, there was no other control to prevent them from saccading towards the targets. Thus, our study could not rule out the potential influence of eye movement on VWM performance in different encoding time conditions. Instead, the eye movement could be considered a potential mechanism underlying the two-stage process in VWM consolidation. The early consolidation stage may be the stage before the saccade. At this stage, low-precision VWM representations are formed in an all-or-none way based on the visual attention shift. After saccades toward the targets begin, individuals enter the late consolidation stage. In this stage, high-precision perception representations are created. By gradually allocating and reallocating more VWM resources to targeted perception representations, individuals consolidate visual information to high-precision VWM representations in a coarse-to-fine way. Thus, the eye movement system may help participants complete the two-stage process. The eye movement effect can provide an ecologically valid explanation for the variability of VWM precision under the different encoding time conditions in our study. We are not saying that eye movement is the essential cause of the two-stage process, but the eye movement effect may be a potential complement to the two-stage process hypothesis on VWM consolidation. Surprisingly, most of the previous VWM studies that manipulated the exposure duration of memory array did not consider the effect of eye movement on their results (especially the influence on VWM precision)^9,15,36^. This omission may have led researchers to overlook or underestimate the contribution of the eye movement system to the VWM process. Future studies need to systematically investigate the effect of eye movement (e.g., saccade times, cumulative fixation time) on VWM performance.

A recent study used functional magnetic resonance imaging (fMRI) to investigate the effects of various consolidation times on the underlying brain activation patterns in a VWM task^37^. The results suggested that there was no significant effect of consolidation time on the functional activation patterns. However, according to the two-stage process hypothesis, there should be a significant difference in functional activation patterns between the early and late stages of consolidation. One possible explanation is that the consolidation time in their study was too long. They asked participants to memorize three or five colors and manipulated the consolidation time from 500 to 1,200 ms. This consolidation time was long enough for participants to enter the late consolidation stage. Since participants were in the late stage in all consolidation time conditions, it is reasonable to suggest that there was no difference in the functional activation patterns across conditions. Therefore, in the future, it will be necessary to manipulate the consolidation time across a broader range to observe differences in functional activation patterns for the two-stage process during VWM consolidation. Moreover, there were differences in the processing of orientation materials and color materials in VWM resource allocation^38,39^. For example, Liu and Becker^6^ found that the consolidation of orientation items was a serial process. Recent studies have found that participants consolidated color materials in a parallel manner^4–6^. Thus, future studies ought to use color materials to test the two-stage process hypothesis.

## Data Availability

The datasets generated during and analyzed during this study are available from the corresponding author (lq780614@163.com, Qiang Liu) upon reasonable request.
